# Dynamic microbial populations along the Cuyahoga River

**DOI:** 10.1371/journal.pone.0186290

**Published:** 2017-10-19

**Authors:** Matthew V. Cannon, Joseph Craine, James Hester, Amanda Shalkhauser, Ernest R. Chan, Kyle Logue, Scott Small, David Serre

**Affiliations:** 1 Cleveland Clinic, Genomic Medicine Institute, Cleveland,OH, United States of America; 2 Jonah Ventures, Manhattan, KS, United States of America; 3 Case Western Reserve University, Department of Global Health and Diseases, Cleveland, OH, United States of America; Oklahoma State University, UNITED STATES

## Abstract

The study of the microbial communities has gained traction in recent years with the advent of next-generation sequencing with, or without, PCR-based amplification of the 16S ribosomal RNA region. Such studies have been applied to topics as diverse as human health and environmental ecology. Fewer studies have investigated taxa outside of bacteria, however. We present here data demonstrating the utility of studying taxa outside of bacteria including algae, diatoms, archaea and fungi. Here, we show how location along the Cuyahoga River as well as a transient rainfall event heavily influence the microbial composition. Our data reveal how individual OTUs vary between samples and how the patterns of OTU abundance can accurately predict sampling location. The clustering of samples reveals that these taxa are all sensitive to water conditions in unique ways and demonstrate that, for our dataset, algae was most distinctive between sample groups, surpassing bacteria. Diversity between sampling sites could allow studies investigating pollution or water quality to identify marker OTUs or patterns of OTU abundance as indicators to assess environmental conditions or the impact of human activity. We also directly compare data derived from primers amplifying distinct taxa and show that taxa besides bacteria are excellent indicators of water condition.

## Introduction

Human activities can dramatically affect the environment. For instance, water quality and aquatic ecosystem health are influenced by pollutants and other environmental disturbances. Given how frequently waterways are exposed to industrial waste or runoff from agricultural or residential areas, the monitoring and preservation of the health of our waterways is a critical undertaking [1–3]. There are many ways in which aquatic ecosystems and water quality are assessed, including both abiotic and biotic indicators such as the detection of specific chemical compounds or species in a monitored area [4,5]. The quantification of sensitive species is a commonly used method to assess the health of a waterway. These species can be either macro- or micro-organisms and their absence, presence or abundance provides important information regarding ecosystem condition. The composition of microorganism communities within a water sample in particular could provide excellent means of assessing water quality and increasingly, the study of microbial composition has become a very active field of research [5,6]. Most of the research done has focused on the bacterial composition within samples, primarily through sequencing portions of the 16S ribosomal RNA to identify species [7–12]. However, the analysis of bacteria investigates only a portion of the microbial community within an aquatic ecosystem. Other taxa are understudied with relatively few publications, though previous reports have investigated the biodiversity of individual taxa in marine and freshwater environments including algae and diatoms [13–16], archaea [17–19] and fungi [20].

To highlight and evaluate the utility of data derived from primers amplifying bacteria, archaea, algae, diatoms or fungi we analyzed data provided with a previous publication [21] describing environmental DNA (eDNA) samples from the Cuyahoga River (Ohio, USA). We analyzed these datasets to determine two things. First, we wanted to examine how different the sample groups were with regards to the microbial composition and determine if these differences were adequate to group the samples. This would reaffirm the utility of non-bacterial datasets for the evaluation of water conditions. Secondly, we wanted to compare these datasets generated from identical samples together to determine which taxon appears to be most sensitive to water conditions. This information could inform future studies evaluating the microbial composition of aquatic environments.

Using the data from our previous publication [21], we here show how algae, diatoms, fungi, archaea and bacteria are all sensitive indicators of water conditions based on their microbial composition. Organisms identified using DNA barcoding can discriminate samples taken from three portions of the Cuyahoga River at two time points. Additionally, we show that principal component analysis can easily detect the dramatic effect of rainfall on the microbial content of a river. These analyses also identify species that vary the most between water samples. Overall, this information could help identify marker species or investigate the biological role of these species in responding to water condition or quality.

## Methods

Data for these analyses are taken from the publicly available SRA database (accession #SRP058316) and details on data generation were previously published [21]. Briefly, we collected surface water by hand in a 50ml conical tube one meter from the bank along three sections of the Cuyahoga River on two separate days. No specific permissions to collect the samples was required as no live animals were collected or affected. No endangered or protected species were directly involved with the collection of the samples. We took the samples back to the laboratory and centrifuged 40ml of water for 30 minutes at 8,000 x g at 4°C to pellet all particulate matter (which could include silt, organic matter, small organisms, etc.) and then froze the samples until DNA isolation. We isolated DNA using the DNeasy blood and tissue kit (Qiagen) and used a two-step PCR protocol to amplify and barcode all samples using taxa specific primers (available as S2 Table at https://images.nature.com/original/nature-assets/srep/2016/160311/srep22908/extref/srep22908-s1.pdf). For these analyses, we excluded any sample from the original study from external sources entering the river. We generated bacterial 16S sequences from the same samples at the same time, using primers modified from previous publications (CTTTCCCTACACGACGCTCTTCCGATCTAGYGGCGIACGGGTGAGTAA [22] and GTGACTGGAGTTCAGACGTGTGCTCTTCCGATCTTGCTGCCTCCCGTAGGAGT [23]) using the PCR protocol described [21]. We previously defined four groups of samples based on location along the river and if the sampling was performed before or after the major rain event described in the original publication [21]: upper river before the rain (upper before, n = 23), upper river after the rain (upper after, n = 15), middle river before the rain (middle before, n = 10) and lower river after the rain (lower after, n = 22). We detail sampling groups and locations in S1 Table.

### QIIME analysis

To analyze DNA sequences (bacteria, archaea, algae, diatoms and fungi) we used the algorithms implemented in QIIME (version 1.8.0) [24]. We analyzed all datasets identically aside from the database used for OTU picking. For bacteria and archaea, we used the Greengenes 16S rRNA database (gg_13_8_otus, May, 2013) [25] and used a 97% sequence identity cutoff to define operational taxonomic units (OTUs). To analyze DNA sequences amplified by the algal, diatom and fungal primers, we used the uclust algorithm implemented in QIIME to define OTUs by grouping together reads with more than 95% nucleotide identity. We then blasted the representative sequence from each OTU against the nt database, retrieved the taxonomic information of the best hit(s) and used custom bash and perl scripts (all code available at https://github.com/MVesuviusC/Cuy2Analysis) to eliminate sequences with off-target hits (i.e., sequences not within the targeted taxon and unknown sequences with less than 80% identical to any known sequence). The primers used amplified species outside of the desired taxa. By clustering OTUs from our reads and keeping only on-target OTUs we maintain all on-target reads during OTU picking while excluding all off-target OTUs from the analysis within each dataset.

We then assigned the reads to OTUs using QIIME’s pick_closed_reference_otus.py script. We used biom summarize-table to determine read subsampling levels for each dataset. We subsampled each dataset within the beta_diversity_through_plots.py script and converted the output biom table to tab delimited with biom convert using the—biom-to-classic-table option. For further analyses, we separately subsampled each dataset within QIIME to both the same level across all datasets (350 reads / sample) and to a higher level as appropriate (16S rRNA bacteria: 20,000, 16S rRNA archaea: 2,000, 23S rRNA algae: 600, 18S rRNA diatom: 1,400, ITS fungus: 350).

### Principal component analysis

We used the subsampled OTU tables produced by QIIME as input for principal component analysis in R. We removed any OTU representing less than 1% of the total reads in all samples to remove OTUs with very few reads. We scaled the read count values within the prcomp() function and calculated the principal components. We omitted obvious outliers from each dataset and recalculated principal components. We tested for differences between groups for PC1-3 using pairwise Students t-tests with a Bonferroni correction for multiple testing. We plotted principal components as both scatterplots and boxplots using the ggplot2 [26], and gridExtra [27] R packages. We extracted the loadings values from the principal component analysis and matched taxonomic information to each OTU.

To quantify how well PC1-3 separated our groups of samples we used the principal component values to perform k-means clustering, assigning the samples to one of four groups with no a-priori information about the sample groups. We then calculated the accuracy with which the k-means clusters recapitulated the actual groups. We did this by beginning with the largest k-means cluster and assigning that cluster to the sample group with the largest representation. Any samples within the cluster from other groups were counted as mis-assigned. We repeated this process for each cluster in turn, from largest to smallest. If the most numerous sample group within a cluster had already been assigned to a larger cluster, the next most frequent group was assigned instead. We divided the total number of mis-assigned samples by the total number of samples to calculate the accuracy of the clustering. For instance, if all groups were assigned to exclusive clusters such that no samples were mis-assigned, the accuracy was 100%. If half of the samples were mis-assigned, the accuracy was 50%. To calculate the likelihood of groupings happening by chance, we randomly redistributed the cluster assignments 1,000 times and calculated the accuracy of assignment for each permutation. We calculated the proportion of random permutations more accurately recapitulating the known groups as a p-value. The results were plotted using ggplot2 in R [26].

We also merged all data from each dataset and performed PCA. We estimated the accuracy of the recapitulation of groupings using k-means cluster analysis.

### Testing for differences in OTU abundance between sample groups

We tested for differences in abundance of individual OTUs between sample groups using pairwise Student’s t-tests with a Bonferroni multiple testing correction.

## Results

We reanalyzed here DNA sequences we had previously generated (accession # SRP058316) from water samples collected along the Cuyahoga River [21]. We generated a total of 10,808,663 paired-end reads of 250 bp for the bacterial 16S rRNA amplification products (S1 Table). All other datasets combined had 8,584,793 reads. Archaea had 1,249,457 reads, fungi had 788,420 reads, algae had 370,625 reads and diatoms had the fewest with 302,824 reads (S1 Table).

To account for the complexity of microorganisms present in the Cuyahoga River and the limited number of annotated sequences available for many taxa, we used QIIME [24] to define operational taxonomic units (OTUs) rather than try to identify species. After OTU assignment, we discarded reads amplified from organisms outside of the targeted taxa and analyzed 38.5%-92.6% of the reads for each dataset (S1 Table). Off target amplification for 23S primers included plants, cyanobacteria and Euglenida. The diatom primers had off target amplification of non-diatom algae, molds and fungi. Fungal primers mis-amplified a wide variety of organisms including plants, insects, arachnids and fish. To account for variations in numbers of reads generated for each sample, we randomly subsampled the same number of reads from each dataset for each sample. We defined 7,450 OTUs of bacteria (16S rRNA primers), 486 OTUs of archaea (16S rRNA primers), 2,069 OTUs of algae (23SrRNA primers), 3,275 OTUs of diatoms (18S rRNA primers) and 1,065 OTUs of fungi (ITS primers). To remove lowly informative OTUs, we discarded all OTUs representing less than 1% of the reads across all samples. We analyzed 106 OTUs for algae (5% of OTUs representing 74% of the reads), 88 for bacteria (1% of OTUs representing 62% of the reads), 147 for archaea (31% of OTUs representing 96% of the reads), 82 for diatoms (3% of OTUs representing 77% of the reads) and 267 for fungi (25% of OTUs representing 92% of the reads).

For the bacteria, the top ten OTUs (by total number of reads, Table 1) were all from the *Betaproteobacteria* class, and primarily of the *Burkholderiales* order, though the most numerous OTU was of the order *Rhodocyclales*. The families represented included *Rhodocyclaceae*, *Comamonadaceae* and *Oxalobacteraceae*. Comparing the 88 OTUs in the dataset (by t-test with a Bonferroni correction), 84 (95%) had different relative abundance between at least two sample groups (p < 0.05, S2 Table).

**Table 1:** Top ten OTUs by total read count across all samples per dataset.

Kingdom	Phylum	Class	Order	Family	Genus and Species	Blast hit percent identity	Reads per OTU	Dataset
*Bacteria*	*Proteobacteria*	*Betaproteobacteria*	*Rhodocyclales*	*Rhodocyclaceae*	*C39*	ND	61,248	Bacteria
*Bacteria*	*Proteobacteria*	*Betaproteobacteria*	*Burkholderiales*	*Comamonadaceae*	*NA*	ND	56,539	Bacteria
*Bacteria*	*Proteobacteria*	*Betaproteobacteria*	*Burkholderiales*	*Comamonadaceae*	*Limnohabitans*	ND	54,720	Bacteria
*Bacteria*	*Proteobacteria*	*Betaproteobacteria*	*Burkholderiales*	*Comamonadaceae*	*NA*	ND	34,661	Bacteria
*Bacteria*	*Proteobacteria*	*Betaproteobacteria*	*Burkholderiales*	*Oxalobacteraceae*	*Polynucleobacter*	ND	29,309	Bacteria
*Bacteria*	*Proteobacteria*	*Betaproteobacteria*	*Burkholderiales*	*Comamonadaceae*	*Limnohabitans*	ND	24,365	Bacteria
*Bacteria*	*Proteobacteria*	*Betaproteobacteria*	*Burkholderiales*	*Comamonadaceae*	*NA*	ND	23,701	Bacteria
*Bacteria*	*Proteobacteria*	*Betaproteobacteria*	*Burkholderiales*	*Oxalobacteraceae*	*NA*	ND	21,135	Bacteria
*Bacteria*	*Proteobacteria*	*Betaproteobacteria*	*Burkholderiales*	*Comamonadaceae*	*Limnohabitans curvus*	ND	19,377	Bacteria
*Bacteria*	*Proteobacteria*	*Betaproteobacteria*	*Burkholderiales*	*Oxalobacteraceae*	*NA*	ND	18,334	Bacteria
*NA*	*NA*	*Cryptophyta*	*Cryptomonadales*	*Cryptomonadaceae*	*Cryptomonas curvata*	98.5	2,183	Algae
*NA*	*NA*	*Cryptophyta*	*Cryptomonadales*	*Cryptomonadaceae*	*Cryptomonas marssonii*	98.5	2,129	Algae
*NA*	*Bacillariophyta*	*Coscinodiscophyceae*	*NA*	*Thalassiosiraceae*	*Thalassiosira pseudonana*	98.8	1,601	Algae
*NA*	*NA*	*Cryptophyta*	*Cryptomonadales*	*Cryptomonadaceae*	*Cryptomonas marssonii*	96.1	1,468	Algae
*NA*	*NA*	*Cryptophyta*	*Pyrenomonadales*	*Chroomonadaceae*	*Chroomonas sp*. *HBI 3577x*	97.9	1,405	Algae
*NA*	*NA*	*Cryptophyta*	*Cryptomonadales*	*Cryptomonadaceae*	*Cryptomonas marssonii*	94.9	1,355	Algae
*NA*	*Bacillariophyta*	*Bacillariophyceae*	*Naviculales*	*Naviculaceae*	*Fistulifera pelliculosa*	95.5	1,088	Algae
*NA*	*NA*	*Cryptophyta*	*Cryptomonadales*	*Cryptomonadaceae*	*Plagioselmis nannoplanctica*	96.7	1,028	Algae
*NA*	*Bacillariophyta*	*Coscinodiscophyceae*	*Melosirales*	*Melosiraceae*	*Melosira varians*	99.7	884	Algae
*NA*	*Bacillariophyta*	*Bacillariophyceae*	*Naviculales*	*Naviculaceae*	*Navicula sp*. *BOLD*:*AAO6950*	100.0	787	Algae
*Archaea*	*Euryarchaeota*	*Methanobacteria*	*Methanobacteriales*	*Methanobacteriaceae*	*Methanobrevibacter*	ND	17,800	Archaea
*Archaea*	*Euryarchaeota*	*Methanomicrobia*	*Methanomicrobiales*	*Methanospirillaceae*	*Methanospirillum*	ND	13,958	Archaea
*Archaea*	*Crenarchaeota*	*MBGB*	*NA*	*NA*	*NA*	ND	11,221	Archaea
*Archaea*	*Crenarchaeota*	*MCG*	*pGrfC26*	*NA*	*NA*	ND	10,261	Archaea
*Archaea*	*Euryarchaeota*	*Thermoplasmata*	*E2*	*DHVEG-1*	*NA*	ND	9,462	Archaea
*Archaea*	*Euryarchaeota*	*Methanomicrobia*	*Methanosarcinales*	*ANME-2D*	*NA*	ND	5,755	Archaea
*Archaea*	*Crenarchaeota*	*Thaumarchaeota*	*Nitrososphaerales*	*Nitrososphaeraceae*	*Candidatus Nitrososphaera*	ND	4,693	Archaea
*Archaea*	*Euryarchaeota*	*Thermoplasmata*	*E2*	*DHVEG-1*	*NA*	ND	4,625	Archaea
*Archaea*	*Crenarchaeota*	*Thaumarchaeota*	*Cenarchaeales*	*Cenarchaeaceae*	*Nitrosopumilus*	ND	4,444	Archaea
*Archaea*	*Crenarchaeota*	*Thaumarchaeota*	*Nitrososphaerales*	*Nitrososphaeraceae*	*Candidatus Nitrososphaera*	ND	3,436	Archaea
*Fungi*	*Basidiomycota*	*Tremellomycetes*	*Tremellales*	*NA*	*Bullera ninhbinhensis*	80.1	992	Fungus
*Fungi*	*Ascomycota*	*Dothideomycetes*	*Capnodiales*	*Mycosphaerellaceae*	*Zymoseptoria verkleyi*	98.8	923	Fungus
*Fungi*	*Ascomycota/NA*	*Dothideomycetes/NA*	*Pleosporales/NA*	*Didymellaceae/NA*	*Phoma sp*. *1LA47-3/ Phoma sp*. *MS-2011-F35/ Phoma sp*. *KY-1/ Epicoccum sp*. *JJP-2009a/ Peyronellaea pomorum/ Peyronellaea glomerata/ Peyronellaea calorpreferens/ Phoma sp*. *W21/ Phoma macrostoma/ Phoma sp*. *DS1wsM30b/ Phoma sp*. *H39/ Phoma sp*. *F2/ Phoma aliena/ Phoma sp*. *C6a10/ Phoma sp*. *MS-2011-F26/ fungal endophyte/ Peyronellaea americana/ Phoma herbarum/ Phoma sp*. *1 TMS-2011/ ascomycete sp*. *Vega374*	99.4	916	Fungus
*Fungi*	*Basidiomycota*	*Tremellomycetes*	*Tremellales*	*NA*	*Cryptococcus sp*. *CBS 12710*	86.6	820	Fungus
*Fungi*	*NA*	*NA*	*NA*	*NA*	*fungal sp*. *NLEndoHerit_029_2008N4-30-2C*	96.6	742	Fungus
*Fungi*	*Basidiomycota*	*Tremellomycetes*	*Tremellales*	*NA*	*Cryptococcus sp*. *LH42/ Cryptococcus flavus/ Tremellales sp*. *LM490*	97.2/97.9	725	Fungus
*Fungi*	*Ascomycota*	*NA*	*NA*	*NA*	*Anguillospora longissima*	89.2	715	Fungus
*Fungi*	*NA*	*NA*	*NA*	*NA*	*fungal sp*. *112m*	98.9	673	Fungus
*Fungi*	*Ascomycota*	*Sordariomycetes*	*Calosphaeriales*	*Calosphaeriaceae*	*Togninia sp*. *CBS 122684*	98.0	667	Fungus
*Fungi*	*Ascomycota*	*Sordariomycetes*	*Calosphaeriales*	*NA*	*Calosphaeriales sp*. *ICMP 17422*	88.6	627	Fungus
*NA*	*NA*	*Chrysophyceae*	*Chromulinales*	*Chromulinaceae*	*Uroglena sp*. *CCMP2768*	95.5	21,333	Diatom
*NA*	*NA*	*Chrysophyceae*	*NA*	*NA*	*Spumella-like flagellate JBAF33*	100.0	18,108	Diatom
*NA*	*NA*	*Chrysophyceae*	*Chromulinales*	*Chromulinaceae*	*Uroglena sp*. *CCMP2768*	95.3	7,941	Diatom
*NA*	*NA*	*Chrysophyceae*	*Chromulinales*	*Chromulinaceae*	*Uroglena sp*. *CCMP2768*	96.5	2,857	Diatom
*NA*	*NA*	*Chrysophyceae*	*Chromulinales*	*Paraphysomonadaceae*	*Paraphysomonas butcheri*	96.0	2,203	Diatom
*NA*	*NA*	*Chrysophyceae*	*NA/Chromulinales*	*NA/Chromulinaceae*	*Spumella-like flagellate JBC31/ Spumella-like flagellate JBAS38/ Spumella-like flagellate JBAF32/ Spumella-like flagellate JBC30*	100.0	1,749	Diatom
*NA*	*Bacillariophyta*	*Fragilariophyceae*	*Fragilariales*	*Fragilariaceae*	*Fragilariaceae sp*. *RCC2043*	80.6	1,732	Diatom
*NA*	*NA*	*Chrysophyceae*	*Chromulinales*	*Chromulinaceae*	*Spumella-like flagellate JBNA46*	94.5	1,489	Diatom
*NA*	*NA*	*Chrysophyceae*	*NA*	*NA*	*Chrysophyceae sp*. *CCMP2296*	95.8	1,398	Diatom
*NA*	*NA*	*Chrysophyceae*	*Chromulinales*	*Paraphysomonadaceae*	*Paraphysomonas bandaiensis*	97.3	1,285	Diatom

For archaea, the top ten OTUs (Table 1) included the classes *Methanomicrobia*, *MCG*, *Thaumarchaeota* and *Thermoplasmata* within which are the orders *Methanobacteriales*, *Methanomicrobiales*, *pGrfC26*, *E2*, *Methanosarcinales*, *Nitrososphaerales* and *Cenarchaeales*. The families in the dataset included *Methanobacteriaceae*, *Methanospirillaceae*, *DHVEG*-1, *ANME*-2D, *Nitrososphaeraceae* and *Cenarchaeaceae*. Out of the top ten OTUs, eight had different abundance between at least two sample groups, and 41 of a total of 147 OTUs (27.9%) were different between at least two sample groups (p < 0.05, S3 Table).

For algae, diatoms and fungi datasets, that are less completely represented in the database, each OTU was named according to the taxon of the most similar sequence (> = 80% identity), providing an estimation of what the OTU may be. For the algae dataset, the classes *Cryptophyta*, *Coscinodiscophyceae* and *Bacillariophyceae* made up the top ten OTUs (Table 1). These included the orders *Cryptomonadales*, *Pyrenomonadales*, *Naviculales* and *Melosirales* and the families *Thalassiosiraceae*, *Chroomonadaceae*, *Naviculaceae* and *Melosiraceae*. For algae, nine of the top ten OTUs differed in relative abundance between at least two sample groups, and 89 of the 106 OTUs (84.0%) tested were different between at least two sample groups (p < 0.05, S4 Table).

For the diatom dataset, the classes *Chrysophyceae* and *Fragilariophyceae* made up the top ten OTUs (Table 1). The orders *Chromulinales* and *Fragilariales* as well as the families *Paraphysomonadaceae*, *Chromulinaceae* and *Fragilariaceae*. Of the top ten OTUs, six differed in relative abundance between at least two sample groups and when considering all 82 OTUs, 42 (51.2%) were different between at least two sample groups (p < 0.05, S5 Table).

For the fungal dataset, the classes *Tremellomycetes*, *Dothideomycetes* and *Sordariomycetes* made up the top ten OTUs (Table 1). These OTUs included the orders *Calosphaeriales*, *Capnodiales*, *Pleosporales* and *Tremellales* as well as the families *Mycosphaerellaceae*, *Didymellaceae* and *Calosphaeriaceae*. For the fungal dataset, eight of the top ten OTUs differed in relative abundance between at least two sample groups and 24 of 267 total OTUs (9.0%) differed (p < 0.05, S6 Table).

**Fig 1:**
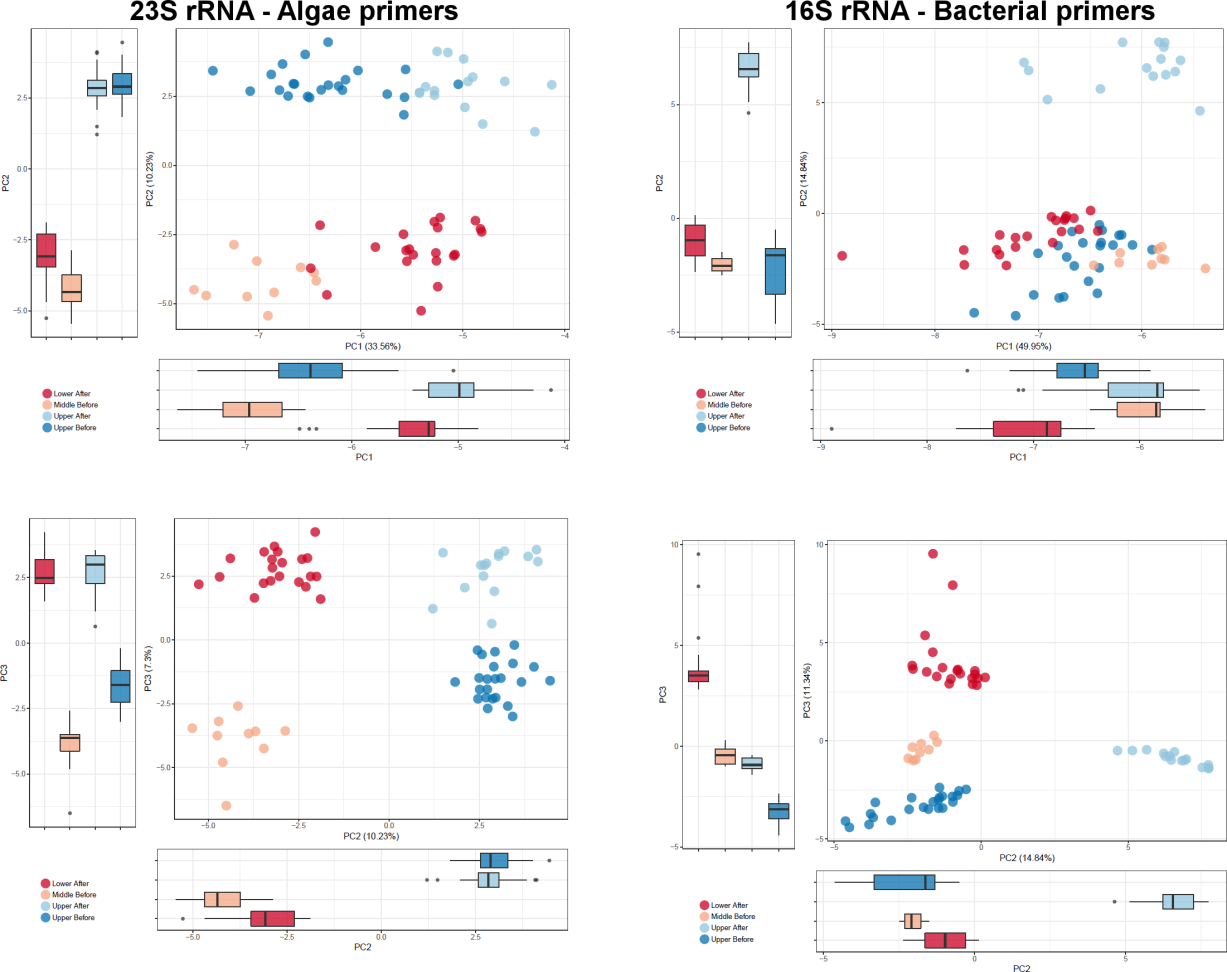
Algae and bacterial PCA analyses. PCA analysis of algal and bacterial datasets reveal that the four sample groups produce distinct sample groups. PC1 vs PC2 and PC2 vs PC3 are presented for both datasets.

We used principal component analysis (PCA) to determine the relationships among the microflora of the samples from four groups (upper Cuyahoga before the rain, upper Cuyahoga after the rain, middle Cuyahoga before the rain and lower Cuyahoga after the rain) and test if some taxa effectively differentiated the groups. Our k-means clustering based analysis outlined in the methods helped estimate how well each PCA analysis discriminated between the portions of the river using the first three principal components. Each dataset had samples assigned to exclusive groups with varying accuracy, with the bacterial and algae datasets demonstrating 100% accuracy (p < 0.001) (Fig 1). The other three datasets were 32.4% (fungus, p = 0.27), 50.7% (archaea, p < 0.001) and 85.9% (diatom p < 0.001) accurate in clustering the actual groups.

The principal component analysis revealed that for each dataset the principal components explain different factors describing the samples. For instance, in the algal dataset, PC1, which explains the most variance within a dataset, separated the sample groups collected before the rain from the two groups collected after the rain (p = 4.5x10^-7^–4.2x10^-14^). In this dataset, the second and third PCs further separated the samples according to their group (Fig 1) resulting in discrete clusters. Indeed, k-means clustering of these samples using the first three principal components yielded a 100% assignment accuracy. For the other datasets, PC1 did not divide the sample groups from before and after the rain. This shows how the sample groups cluster in unique ways for each dataset, indicating that each taxon responds differently to the environments along the river. Another example is the fungal dataset, where none of the three first principal components divide the sample groups from before and after the rainfall (p > = 0.12). This contrasts with the other datasets where the upper sample groups from before or after the rain were separated by at least one principal component. PC2 from the fungal dataset separated the two sample groups from the upper Cuyahoga from the lower and middle portions (p = 2.3x10^-3^–5.5x10^-5^) showing that the fungal composition of the samples did change along the river (S1 Fig). Note that despite differences from the algal dataset, the three first PCs of the bacterial dataset are sufficient to correctly assign all samples to their groups (100% accuracy). On the other hand, for the fungus, archaea, and diatom the OTUs abundance information is not entirely sufficient as they only allow assignment with 32.4%, 50.7% and 85.9% accuracy, respectively.

**Fig 2:**
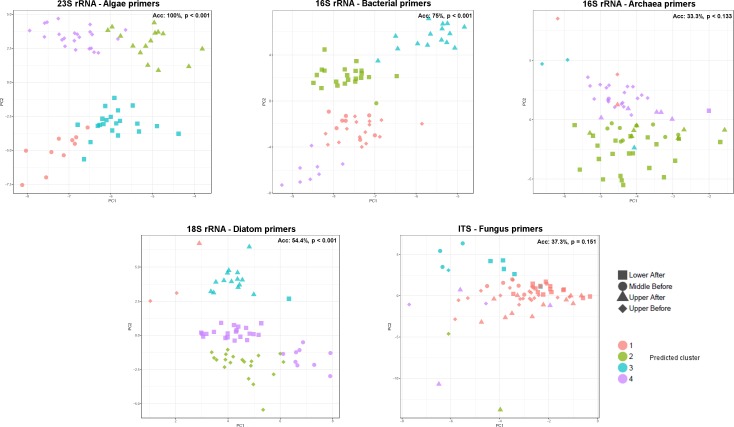
PCA analysis on evenly subsampled datasets. To compare how well each dataset distinguished sample groups, we subsampled each to the same number of reads per sample and performed PCA. The algae dataset was best able to separate the sample groups as shown by the k-means clustering analysis accuracy of 100% compared to the other datasets which had lower accuracies.

Since different numbers of reads were used for each dataset, we tested whether the differences in assignment accuracy between datasets was caused by the amount of information generated or genuine differences in discriminating power of different taxa. We therefore randomly subsampled 350 reads from each dataset per sample within the analysis with QIIME and recalculated the principal components. We then estimated how well each dataset differentiated between sample groups using the k-means clustering analysis described in the methods. We confirmed our primary analyses and found that algae dataset separated the groups the most effectively (accuracy of 100%) (Fig 2). The other taxa separate the groups less efficiently (bacteria: 75% (p < 0.001), diatom: 54.4% (p < 0.001), archaea: 33.3% (p = 0.13) and fungus: 37.3% (p = 0.15)) (Fig 2).

**Fig 3:**
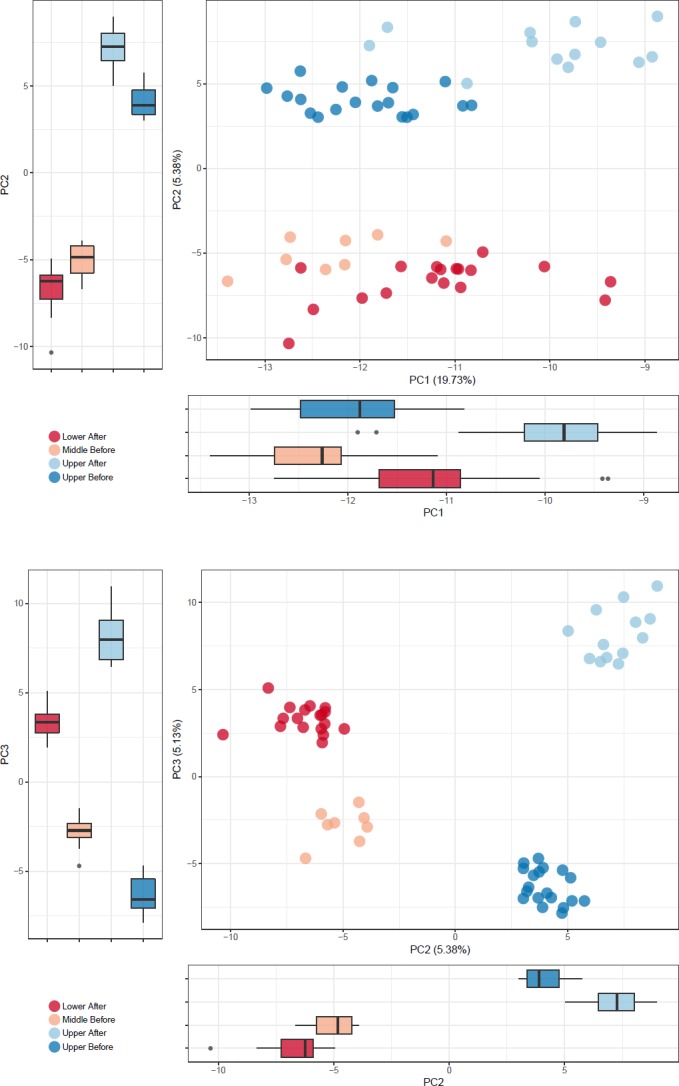
Combined OTU PCA analysis. PCA analysis of OTUs from each dataset combined. OTUs from each dataset were combined into a single dataset to allow comparison of taxa between datasets. PC1 vs PC2 and PC2 vs PC3 are presented.

We then analyzed OTUs from the different datasets together by combining all OTUs and performing PCA. The PCA separated the groups very well, with 100% accuracy (p < 0.001) (Fig 3).

In this PCA including all taxa, PC1 primarily separated samples collected after the precipitation event in the upper Cuyahoga from other sample groups (Fig 3, p = 2.4x10^-3^–6.8x10^-07^). Consistent with the results of the individuals PCAs, the taxa that influenced PC1 were primarily bacteria, algae and diatoms, while fungi and archaea had less influence (S7 Table). Taxa enriched after the precipitation event include bacteria from the family *Comamonadaceae* and *chrysophytes* from *Chromulinaceae* such as *Uroglena sp*. In contrast, chrysophytes from the order Chromulinales most closely related to Spumella species and other bacterial OTUs of the *Comamonadaceae* family were enriched before the rain.

In the all-taxa PCA, PC2 primarily separated samples based on their geographic location (Fig 3, p < 2x10^-16^). Taxa that were more abundant in upper part of the river independent of the precipitation event included a mixture of *chrysophytes*, fungi, archaea, bacteria, and algae (S7 Table). Taxa that best represented upper portion included *Microbacteriaceae* such as *Clavibacter* and *Candidatus*, and eukaryotes such as *Cryptomonas*, *Spumella*, and *Chroomonas*. Taxa representative of lower reaches primarily included bacteria from *Burkholderiales* and *Rhodocyclales*, but also diatoms with sequences similar to *Thalassiosira pseudonana*.

PC3 primarily separated sites collected before the rain vs. those collected after the rain. Bacterial taxa from *Comamonadaceae* and the *chrysophyte Paraphysomonas butcheri* were representative of sites before the precipitation event. Other bacterial taxa from *Rhodocyclaceae* and *Comamonadaceae* as well as eukaryotes such as *Uroglena* and *Cryptomonas* were the primary taxa associated with sites collected after the rain.

We also used this combined analysis to identify correlated OTUs from different taxa. For instance, we found that for PC2, one bacterial OTU of the *Rhodocyclaceae* family correlated with an OTU most similar (95.5% sequence identity) to *Cryptomonas ovata* amplified by the 23S rRNA algae primers, suggesting that similar factors may influence these taxa (S7 Table). However, both OTUs were anticorrelated with a fungal OTU most similar (80.4% sequence identity) to *Pseudoteratosphaeria ohnowa*, indicating that different samples contain these species (S7 Table).

**Fig 4:**
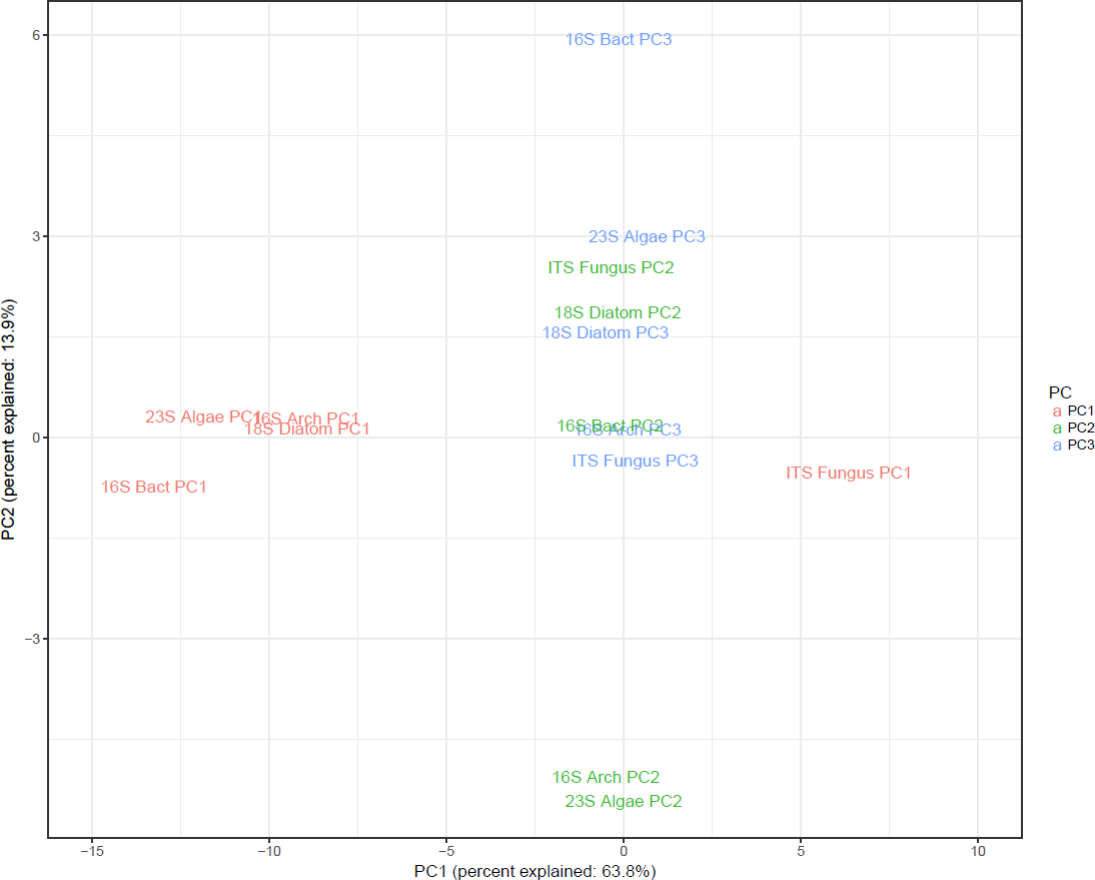
PCA on PC1-3 from each dataset. Principal component analysis on PC1-3 from each dataset. This analysis determined which principal components organize samples from different datasets similarly. The plot shows that PC1 from the algae, bacteria, archaea and diatom datasets are similar and distinct from PC1 in the fungal dataset. PC2 and 3 are similar across all datasets as demonstrated by similar values for PC1, which in this analysis explains 63.8% of the total variance.

We also wanted to see if the principal components from distinct datasets tended to organize samples similarly. To do this, we combined the principal component values of the first three principal components from each sample in each dataset. We then performed principal component analysis on these values. We found that PC1 separated the samples similarly for the bacterial, algal, archaeal and diatom datasets (Fig 4). PC1 from the fungal dataset separated the samples in a distinct pattern as demonstrated by its separation from the other PC1 dataset values. PC2-3 did not form clusters like PC1 indicating that each dataset responded uniquely (Fig 4).

## Discussion

Using data from the SRA database, we evaluated how well amplicon sequencing of diverse taxonomic groups can differentiate between portions of the Cuyahoga River. The sequences represent one full MiSeq run (bacterial dataset) and a portion of a second MiSeq run (all other datasets). This led to considerable differences in the number of reads included in each dataset and the number of OTUs represented. Despite each dataset being subsampled to a low number of reads (aside from bacteria) during the analysis, we were still able to describe considerable diversity between samples taken along the Cuyahoga River.

We wanted to examine two primary questions using these data. We wanted to see if each sample group had distinct microbial communities. We also wanted to compare the datasets using equal read counts to see which taxa was most sensitive to varying river conditions.

Our principal component analysis showed the microbial diversity between portions of the river very well. For most datasets, the four groups of samples formed distinct groupings. Differences in patterns between datasets, however, highlight the utility of using multiple taxa to evaluate environmental water samples. Each assayed taxonomic group responded to the conditions along the river in distinct ways as evidenced by the different PCA clustering patterns. For instance, algae seemed to be very heavily influenced by rainfall as the before samples and after samples clustered together along PC1 (Fig 1). Factors unquantified in this study such as water temperature or weather patterns may have influenced microbial diversity between the two sampling time points. Further study is required to confirm the effect of rain on microbial composition of a river though our data suggest that any study investigating aquatic microbial diversity should consider rainfall as a potential influencing factor. Bacteria seemed less affected as the sample groups collected before the rain did not separate from the samples taken after the rain for the first principal component. This suggests that the river algal community changes more after rainfall than the bacterial community does. This could be due to factors such as sensitivity to pH or salinity or even a change in the rate of influx of taxa from nearby terrestrial sources. Our data highlight how investigation of multiple taxonomic groups can reveal differences in response to environmental factors and that taxa outside of bacteria can provide important insight into the interplay between water conditions and microbial communities. For instance, our PCA analysis performed on the PC values for each dataset shows that all datasets except the fungal are most influenced by similar factors as demonstrated by the clustering of the individual PC1 values (Fig 4). For a study collecting data on specific abiotic or chemical concentrations across sampling locations, the differences in microbial diversity along the river could inform studies on the biological impact of these factors.

The datasets analyzed initially had very different quantities of data. If we wish to determine which taxonomic groups were most influenced by the conditions along the Cuyahoga River, we need to equalize the data to avoid bias introduced by differences in the total amount of data. By subsampling each dataset to the same number of reads per sample, we therefore can make direct comparisons between them. This analysis highlights how well the algal dataset performs compared to bacteria. Bacteria segregated the sample groups with only a 75% accuracy (Fig 2) compared to 100% accurate group assignments for algae. It is very important, however, to point out that the comparisons between datasets are comparing the primer sets used to amplify the samples (which likely only amplify a portion of all species within the target taxa) as much as the individual taxonomic groups. Based on these data using only a single primer pair per taxonomic group we cannot conclusively state that, for instance, fungal communities are less dynamic in response to environmental conditions than algae. We can only state that the taxa amplifiable by our primers change less between sample groups. Primers capturing information on a different and/or broader group of fungal species may have produced a very different result.

Our analysis of the all OTUs from each dataset together provides an interesting perspective on the overall dataset. This analysis allows us to easily observe which taxa between datasets are either correlated or anticorrelated. These correlations could indicate that the species interact in some way, that they thrive best in similar environments, or that they are entering the environment from the same external source. Therefore, these data point towards interesting avenues for future research. For instance, the bacterial OTU of the *Rhodocyclaceae* family and *C39* genus and the algal OTU most closely matching *Cryptomonas ovata* were both highly abundant in the upper Cuyahoga River after the rain and essentially absent before, suggesting that these OTUs may have washed into the river and may not normally inhabit those sampling sites. The OTU most closely matching *Pseudoteratosphaeria ohnowa* is present at high abundance in the lower Cuyahoga after the rain, but essentially absent everywhere else, suggesting that it is either introduced in the lower Cuyahoga, or, the other portions of the river are not suitable habitat for this species.

The identification of specific OTUs that differ between portions of the river can inform environmental studies. For instance, a study using multiple taxa to evaluate water samples could investigate the correlation between biotic and abiotic factors (temperature, nitrates, pH, dissolved O_2_, etc) to find what contributes most to microbial differences. Alternatively, such data could reveal how changes in concentration of abiotic factors in an ecosystem are likely to influence the base of the food chain. This could also help identify which conditions influence biodiversity across different types of environments.

A microbial signature based on DNA from organisms found in the water for locations known to harbor an endangered species could aid in evaluation of the suitability of other habitats for relocation. This could also help locate sites harboring previously undescribed populations.

Overall, this study supports the investigation of water condition and environmental differences using amplicon sequencing targeting many different taxa that respond uniquely to environmental factors. Comparing head to head, the data generated with our 23S rRNA algae primers were the most distinct between sample groups, but the data from the bacterial 16S rRNA primers separated the groups with reasonable accuracy. The other primer sets amplifying diatoms, fungi and archaea also produced quality data, but those taxa did not seem to change compositions as dramatically between sample groups. Future studies will be necessary to determine if this is a consistent result or if it is specific to the taxa amplifiable by our primers. Data such as these combined with measures of abiotic conditions or specific chemicals would provide insight into which taxa are most sensitive to specific aquatic conditions and demonstrate which conditions or chemicals most dramatically alter the environment.

## Supporting information

S1 Fig Archaea, diatom and fungus PCA analyses. PCA analysis of archaea, diatom and fungal datasets reveal that the four sample groups produce distinct sample groups. PC1 vs PC2 and PC2 vs PC3 are presented for both datasets.
(TIFF)

S1 Table Sequencing read numbers. (XLSX)

S2 Table 23S rRNA algae dataset OTU loadings and taxa. (XLSX)

S3 Table 16S rRNA bacteria dataset OTU loadings and taxa. (XLSX)

S4 Table 18S rRNA diatom dataset OTU loadings and taxa. (XLSX)

S5 Table 16S rRNA archaea dataset OTU loadings and taxa. (XLSX)

S6 Table ITS fungus dataset OTU loadings and taxa. (XLSX)

S7 Table OTU loadings and taxa for analysis of all OTUs from each dataset combined. (XLSX)
