# Supplementary figures and images for: Dynamic microbial populations along the Cuyahoga River
Source: PLoS One. 2017 Oct 19;12(10):e0186290. doi: 10.1371/journal.pone.0186290 (PMC5648161; doi:10.1371/journal.pone.0186290)

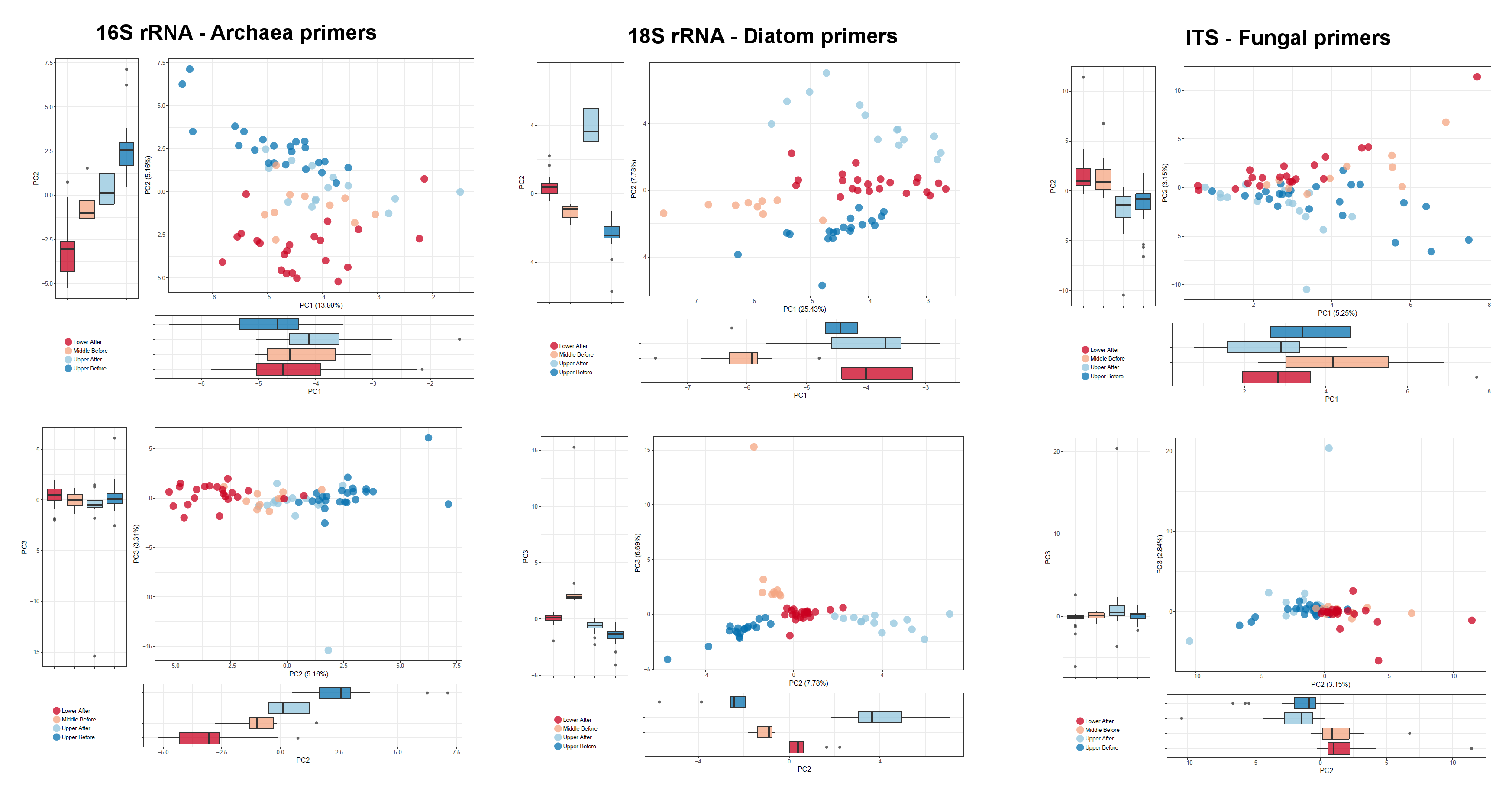

Supplement: S1 Fig — PCA analysis of archaea, diatom and fungal datasets reveal that the four sample groups produce distinct sample groups. PC1 vs PC2 and PC2 vs PC3 are presented for both datasets. (TIFF) [file pone.0186290.s001.tiff]
